# Clinicians’ experiences in signposting an online mental health resource to expectant mothers: a qualitative study

**DOI:** 10.1186/s12884-023-05671-w

**Published:** 2023-05-10

**Authors:** Sofie Saxild, Philip Wilson, Sarah de Voss, Gritt Overbeck

**Affiliations:** 1 The Research Unit for General Practice and Section of General Practice, Department of Public Health, University of Copenhagen, Copenhagen, Denmark; 2 Centre for Rural Health, Institute of Applied Health Sciences, University of Aberdeen, Scotland, United Kingdom

**Keywords:** Preventive care, Web-based intervention, General practice, Mental health, Pregnancy, Primary healthcare, Parent-child relation, Child development

## Abstract

**Background:**

Poor maternal mental well-being and a lack of secure parent-infant attachment, have been identified as important factors associated with adverse mental health outcomes later in a child’s life. Interventions designed to care for maternal mental well-being during pregnancy and early parenthood, are therefore likely to support healthy child development. Mentalization is a skill parents can practice, improving the emotional bond to the child, offering insights into their own and the child’s mental states and potentially improving parental mental well-being. Most pregnant women in Denmark schedule antenatal consultations in general practice, potentially offering a solid platform to promote web-based interventions aiming to enhance mentalization skills. Signposting towards online resources has several advantages including high accessibility, ease of distribution and cost-effectiveness. We aimed to explore the attitudes and experiences of clinicians in general practice in signposting women towards a web-intervention to increase parental mentalization skills.

**Methods:**

The intervention was offered to pregnant women at their primary preventive antenatal consultation in Danish general practice around week eight of pregnancy, and was designed to be incorporated into the following antenatal- and pediatric consultations until the child’s second birthday. Semi-structured interviews about clinicians’ experiences with signposting the intervention were conducted with 11 general practitioners (GP), three practice midwives (MW) and one practice nurse (NR).

**Results:**

Clinicians wanted to enhance the focus on mental well-being in pregnancy and early childhood during preventive consultations. The main barriers to signposting the web-program were decreasing motivation over time, lack of financial viability and time limitations. Utilizing a psychoeducational web-intervention was generally accepted by clinicians, but ideally not carried out solely in general practice.

**Conclusion:**

Signposting web-programs to improve parental mentalization skills can be welcomed by clinicians in general practice but need to be more tailored to suit the everyday workflow of the clinics. Addressing parental mentalization remains largely unchartered territory for pregnant women and clinicians alike, therefore training clinicians on the subject and its presentation should be offered.

**Trial registration:**

The study is part of a larger project that has been approved by the Research Ethics Committee at the University of Copenhagen, Nov. 2019 (reference number 504–0111/19–5000).

## Background

Mental illness is often rooted in childhood [1] and is influenced by genetic, obstetric and environmental factors including parental physical and psychological well-being [2, 3]. The pre- and postnatal period, are highly associated with the risk of deterioration in maternal mental health with adverse consequences for mother and child [4–6]. One key factor in the development of mental resilience is the formation of secure child-parent attachment in early childhood, facilitated in part by good parental mentalization capacity [7]. Mentalization is the ability to understand mental states as explanations for the behavior and reactions of oneself and others, and is an ability that can be improved through training [7]. Optimizing parents’ mentalization abilities could therefore enhance future mental resilience among their children.

Initiatives regarding preventive care during pregnancy and childhood have been established worldwide with the purpose of proactively securing the health of mother and child [8]. Preventive antenatal and pediatric development consultations in Denmark date back to the 1940s and have a high participation rate [9]. GPs perceive these consultations as a pivotal part of their job [10]. The Danish National Board of Health has outlined recommendations for the consultations [11] and GPs are reimbursed for 20 min for each preventive consultation by the health authority, and extra time (20 min for the initial consultation and 10 min for subsequent consultations) was paid for by the FamilieTrivsel project. However, it is up to each individual GP to develop the framework for carrying out the consultations, including delegation to other clinic staff such as nurses or midwives. Positive maternal mental health during pregnancy seems to lower the risk of the child developing mental or behavioral disorders later in life, which makes the subject of maternal mental well-being highly relevant to antenatal consultations [12].

Web-based interventions such as online cognitive behavioral therapy can be effective when treating mental health conditions [13] and could potentially be useful in sustaining mental well-being during pregnancy. Online programs offer several advantages including temporal flexibility, cost-effectiveness and ease of distribution [14]. To improve parental mentalization, a web-based low-cost psychoeducational intervention was developed based on the “Resilience Program” which has been tested in other settings [15]. The web-portal leads to a range of flexible modules offering information about resilience, mentalization, stress-management and cognitive training. A simple set of presentations combining texts, pictures and short narrated soundbites, also functions as a directory for parents about their relationship with the developing child [16, 17].

## Methods

The aim of this study is to explore the attitudes and experience of general practice clinicians who directed their patients to the Resilience Program web-portal.

### Study setting

We conducted qualitative follow-up research nested in a randomized controlled trial testing a parental education program delivered in general practice [16]. General practices from two Danish administrative regions (Capital Region and Zealand) were invited to participate in the “Family Well-being” study in October 2019. Sixty-one general practices joined and were randomized into intervention- and control clinics. Between November 2019 and March 2020 GPs, midwives and nurses from the intervention clinics attended a one-day course where they learned about parental mentalization, were introduced to the intervention and trained to deliver its content to pregnant women. The intervention has been described in detail elsewhere [16].

### Study design

Twenty-eight practices from the intervention group were invited by e-mail from Sofie Saxild (SS) to participate in an interview for this study: 13 clinics accepted, eight never responded and seven declined. The clinics that declined primarily did so because of difficulty finding the time to schedule the interview. Invited clinics were selected based on having the highest number of recruited mothers (10–36), and regional diversity was prioritized. No prior relationship existed between SS and the participants. The 13 interviews comprised 15 clinicians: 11 doctors, three midwives and one nurse. Most interviews were conducted with one GP participant; however, two interviews were conducted with the GP and the clinic midwife or nurse present respectively. Half the GPs preformed psychotherapy to some extent within their consultations, half of the clinicians in total had prior interests in obstetrics and gynecology and a few expressed prior interest in psychiatry. Clinicians’ title, gender and age, as well as clinic location and whether the clinician practiced psychotherapy or mentioned specific interests in psychiatry or gynecology/obstetrics is shown in Table 1.

**Table 1:** Overview of participant and clinic characteristics

Clinician ID	Title	Gender	Age (years) at interview	Clinic location	Practice psychotherapy themselves or were familiar with mentalization	Mentioned specific interests in child- or adult psychiatry	Mentioned specific interests in obstetrics and gynecology
GP-1	Doctor	Male	42	Urban	Yes	No	No
GP-2	Doctor	Female	52	Rural	Yes	Yes	No
GP-3	Doctor	Female	53	Urban	Yes	Yes	No
GP-4	Doctor	Female	42	Urban	Yes	No	Yes
GP-5	Doctor	Female	58	Suburban	No	No	No
GP-6	Doctor	Male	56	Urban	No	No	No
GP-7	Doctor	Female	41	Urban	Yes	No	Yes
GP-8	Doctor	Female	45	Suburban	No	No	Yes
GP-9	Doctor	Female	53	Urban	No	No	No
GP-10	Doctor	Female	47	Urban	Yes	No	Yes
GP-11	Doctor	Female	47	Urban	No	No	No
MW-1	Midwife	Female	58	Urban	No	Yes	Yes
MW-2	Midwife	Female	45	Urban	No	No	Yes
MW-11*	Midwife	Female	57	Urban	No	No	Yes
NR-6**	Nurse	Female	53	Urban	No	No	No

*Is employed at GP-11s general practice and they attended the interview together

**Is employed at GP-6 general practice and they attended the interview together

All participants responded either by e-mail or text message with written informed consent to participate in the study and GPs were reimbursed for their participation.

Data were collected from clinicians through semi-structured interviews between May and June 2021, either in the setting of their practices or by video call. The interview questions were written by SS and approved by all authors.

### Data analysis

Average duration of interviews was 42 min. Interviews were audio recorded and transcribed verbatim by SS and analyzed using the Normalization Process Theory (NPT) framework for studying integration of new practices. The NPT framework consists of four core aspects: clinicians’ sense-making of the new practice; commitment to carrying out the intervention; how workable the new practice is and the clinician’s appraisal of it [18].

Initial analysis and coding of the interview data was performed by SS, and subsequently the work was shared with the co-authors Philip Wilson (PW), Gritt Overbeck (GO) and Sarah de Voss (SV) to review and agree upon.

## Results

Results are presented in the order of the NPT dimensions.

### Making sense of the web-based parental training program

Having a clear understanding of the intervention’s purpose and seeing the benefit of using it as a platform to enhance the focus on mental well-being in the preventive consultations, clinicians sought to integrate the intervention at the first antenatal consultation.

*“I believe that there exists a general lack of focus on mental well-being in families with children – not just in general practice, throughout the healthcare system …” (MW-1)*

*“It was important for me to showcase the website early on and point out that the information it encompassed could not just be Googled.” (GP-2)*

### Clinicians’ commitment to applying the web-based content to the consultations

Clinicians were initially enthusiastic about introducing the website. However,

the level of motivation started to diminish in part because introducing the interventions content was time-consuming.

*“In the beginning we were very motivated … I used the last ten minutes of the first antenatal consultation introducing the project [the intervention] … but it became less and less during the following consultations. Also, because the patients’ interest seemed to decline” (GP-3)*

### Workability of the web-based program in general practice

Overall, clinicians found it feasible to present the intervention in general practice and thought the presentation could be performed by most healthcare professionals in the clinic.

The website served as a conversation starter on the topic of mental health. It allowed the patients to mention psychosocial issues and promoted the clinician-patient alliance.

*“…I sense that patients talk more openly about mental health now, and I believe it is because early on in their pregnancies we articulated the challenges the future might hold, and that in turn made it easier for them to open up about [the issues] later when they actually faced them” (MW-1)*

Since it proved more time-consuming than anticipated to increase the focus on mental well-being during the consultation, in addition to the physical examination, clinicians began doubting that the amount of time available for each consultation was sufficient to go into the online material in depth.

*“… we do not actually use the website in the consultations we only inform the patients that it exists. Displaying and actively exploring the website with the patient during the consultation would be completely impossible, due to time limitations …” (GP-11A)*

Some clinicians felt that first-time mothers showed more interest in the project since those with children viewed themselves as already familiar with mentalization. Additionally, clinicians felt that the mothers participating in the project generally seemed quite resourceful. The thirteen clinics signposted the website to 211 mothers with a mean age of 34,2 years of age (23–52 years). The majority were employed (76%), received standard antenatal care (74,8%), and were almost evenly divided between nulliparous (47,9%) and primiparous (52,1%). Additionally, the mothers were screened using psychometric tests: Hospital Anxiety and Depression scale (HADS) [19], Adverse Childhood Experiences Study (ACE) [20] and Recent Life Events Questionnaire (RLEQ) [21]. Generally, they were considered psychologically robust in ACE and RLEQ but scored with borderline risk of depression and/or anxiety in HADS. Often, when clinicians perceived the mother as resourceful, they tended to introduce and utilize the intervention less thoroughly throughout the consultations. During consultations with mothers who were mentally vulnerable or cognitively challenged clinicians would present the website’s content in layperson’s terms and they sought to engage these mothers more intensely, since it seemed that they struggled more with comprehending and utilizing the websites content.

*“There definitely is a correlation between patients’ cognitive capacity and who used the website. Which is a paradox because I believe that the ones who would benefit the most from learning this, are not equipped with abilities to approach and understand the website” (GP-11A)*

When presenting the website, clinicians suspected that there were incidents where patients felt picked out for discussion about mental health, fearing the clinician viewed them as unstable or found their parenting skills inadequate. Consequently, clinicians strove to relay the content in a sensitive manner, making it approachable, and it became apparent that the clinicians’ approach and phrasing was crucial to the outcome of such conversation.

“*I found it challenging to get the mothers to enroll … The second you mention words like “mental health” and “resilience” they feel almost picked out as bad mothers …” (GP-2)*

### Clinicians’ appraisal of the web-intervention

Most clinicians did ‘buy into’ the intervention and deemed it worthy of endorsement, and they appreciated being able to at least refer patients to the site especially if they were pressed for time during the consultation.

*“the website can most certainly compete with the existing web-based information sites catering to pregnant women and families, because it is much more scientifically based and useful. I feel confident referring my patients to this website and not having them Google all sorts of stuff…” (GP-5)*

Clinicians felt that they had gained a wider range of communication skills and expanded their vocabulary on mental health, and that discussing mental health and family formation was vital in supporting good quality parenting. They strove to discuss these subjects throughout the woman’s pregnancy. The same need for continuity became apparent working with the website since it did not seem sufficient to present it just once; it had to be an integrated part of the consultations if it was to be helpful.

*“This project requires a lot of time, but I think it can be worthwhile if the website is actively used and the patients engage. I am sure many women would benefit from participating … however I do find it exceedingly ambitious when it comes to the amount of time the women actually need to devote to really internalize the theories” (GP-3)*

Clinicians questioned whether the first antenatal consultation around week eight of pregnancy was the most opportune time to introduce the website, wondering whether the knowledge on mentalization would hold up until it was to be used during child rearing.

*“I think it is rather soon to introduce the website at the first antenatal consultation, because in my experience it is not until the final trimester that women start reflecting on motherhood … perhaps all the relevant themes “burn out” throughout the course of the pregnancy if introduced too early…” (MW-1)*

Even though clinicians found it unlikely that time limitations would cease to be a limiting factor in general practice, all believed that increasing the focus on mental well-being in the families should continue, and that the intervention was a useful tool to generate awareness on the matter.

*” …I feel it would be a shame for GPs if they missed out on being part of a movement that focuses on the mental health in families … It would be beneficial to further educate GPs in managing the antenatal- and pediatric consultations when it comes to the psychological aspect” (GP-3)*

## Discussion

Clinicians welcomed the website and found it to have face validity. Several barriers to implantation were found: clinicians felt they had insufficient time to present the content comprehensively. Motivation diminished over time, the intervention seemed to mostly fit the resourceful women and clinicians were concerned that some women felt singled out. Time-limitations during the consultations impeded their opportunity and motivation to include the intervention in the consultations routinely. Some GPs speculated whether the intervention was presented too early and generally found that bringing up mental health could be a delicate matter, but the website could serve as a safe platform to do so.

When attempting to introduce an intervention into any setting, the implementation process can cause disturbances in the organization [22], so to optimize the adoption of the technology, it must be useful and offer up advantages compared to the existing workflows and have early noticeable benefits [23]. Generally, the intervention was deemed useful, but over time clinicians struggled to preserve the feeling of it being advantageous to include in the consultations compared to previous patterns of work. A prominent reason was time-limitations during the consultations which is a major barrier to implementation since new technology should never slow down the users unnecessarily in their daily workflow and should preferably fit existing organizational structures from the beginning [23]. Similar barriers have been described when trying to introduce online therapy interventions in general practice; implementation and follow-up of the intervention was inhibited by factors such as inadequate knowledge of the content, competing workflows of standard treatment and hectic practice [24].

In general, clinicians found the website to be credible, they were competent in accessing and utilizing it and had a positive attitude towards innovation which are all important precursor for successful implementation of new clinical practices [25, 26].

For future web-interventions that promote mental well-being and positive parenting it seems important to ensure continuous involvement and collaboration between the end-users, in this case both mothers and GPs, and the developers throughout all stages, for example offering users to test prototypes to create the best foundation for an implementation [27]. Since web-interventions are an expanding platform, not necessarily comprising solely high-quality products, it is paramount to conduct ongoing evaluation during their development and implementation, while ensuring that leaders in the organization support the new technology by instilling a sense of ownership of the process in their surroundings [28].

Adherence to the intervention is crucial, and even though web-interventions for promoting health and health-related behavior have been proven effective in various areas of healthcare poor adherence is common, which is in line with our findings that adherence declined over time [17, 29, 30].

A major barrier for patients to seek health-related information is a lack of confidence in their capability to correctly understand and apply the information presented [31]. This supports the clinicians’ suspicion that the intervention required a level of literacy too high for mothers with lower cognitive or linguistic abilities. Future web-interventions should maintain focus on optimizing their accessibility to diminish linguistic complexity, perhaps offering various format options for users to choose from, giving mothers the choice of either reducing or increasing the amount of theoretical information presented [31]. For future implementation financial incentives, and correspondingly extra time, should be allocated for this task to be carried out in general practice.

Some GPs consult with mothers of lower socioeconomic status, or mothers where their first language differ from the online material. It might promote acceptability if future web-interventions cater to mothers on a more diverse spectrum, and it would also be more beneficial since lower language skills and poor socioeconomic status are predictors of poor health [32].

### Strengths and limitations

The strengths of this study include the variety of healthcare professionals included in the sample, involved in promoting the intervention. None of the authors were involved in the development of the intervention. The first author (SS) was employed to carry out the interviews well after the intervention had been introduced, and the website applied to the clinics’ consultations, and therefore had no influence on adherence or opinions forming regarding the website. A limitation of the study is that GPs wanting to take part in the project were probably already more inclined to focus on mental well-being in the preventive consultations compared to other GPs, which may influence the implementation if the website was to be rolled out more widely.

## Conclusions

We found that there was interest among clinicians in signposting online tools to help pregnant women and new mothers adapt cognitively and emotionally to their new role, because of the importance of ensuring good mental health early in life. Declining clinician adherence and varying linguistic and cognitive abilities among the mothers, however, were obstacles when trying to implement a long-term meaningful tool to support mental well-being in expectant mothers, in general practice. For future reference when designing a web-portal with a similar aim, it seems important to establish an all-embracing and durable web-based mental health platform, but also to familiarize clinicians with techniques on how to apply and present the content. Health care personnel are open to and interested in digital solutions in the effort to improve mental health for expectant mothers. Successful implementation could yield enormous benefits since it would influence not only mothers but an entire future generation.

## Data Availability

The data that support the findings of this study are available on request from the corresponding author GO. The data are not publicly available since participants in the study did not consent to having the full transcripts of the interviews made publicly available.

## Declarations

### Authors information

First author SS is a medical doctor currently training to become a specialist in family medicine. SS has previously worked in the field of psychiatry. PW and SV are medical doctors as well – PW is an academic general practitioner and researcher, and SV is a PhD student in the field of family medicine. Co-authors PW and GO are well-versed qualitative researchers.

## Abbreviations

GP: General Practitioner
MW: Midwife
NR: Nurse
SS: Sofie Saxild
PW: Philip Wilson
SV: Sarah de Voss
GO: Gritt Overbeck
HADS: Hospital Anxiety and Depression scale
ACE: Adverse Childhood Experiences Study
RLEQ: Recent Life Events Questionnaire

## References

[1] Kessler RC, Berglund P, Demler O, Jin R, Merikangas KR, Walters EE (2005). Lifetime prevalence and age-of-onset distributions of DSM-IV disorders in the National Comorbidity Survey Replication. Arch Gen Psychiatry.

[2] Thompson L, Kemp J, Wilson P, Pritchett R, Minnis H, Toms-Whittle L (2010). What have birth cohort studies asked about genetic, pre- and perinatal exposures and child and adolescent onset mental health outcomes? A systematic review. Eur Child Adolesc Psychiatry.

[3] Puckering C, Allely CS, Doolin O, Purves D, McConnachie A, Johnson PC (2014). Association between parent-infant interactions in infancy and disruptive behaviour disorders at age seven: a nested, case–control ALSPAC study. Bmc Pediatr.

[4] Munk-Olsen T, Pedersen HS, Laursen TM, Fenger-Grøn M, Vedsted P, Vestergaard M (2015). Use of primary health care prior to a postpartum psychiatric episode. Scand J Prim Health Care.

[5] Munk-Olsen T, Maegbaek ML, Johannsen BM, Liu X, Howard LM, di Florio A, Bergink V, Meltzer-Brody S. Perinatal psychiatric episodes: a population-based study on treatment incidence and prevalence. Transl Psychiatry. 2016;6(10):e919. 10.1038/tp.2016.190 PMC5315550 27754485

[6] Munk-Olsen T, Laursen TM, Pedersen CB, Mors O, Mortensen PB (2006). New Parents and Mental Disorders: a Population-Based Register Study. JAMA.

[7] Luyten P, Nijssens L, Fonagy L, Mayes LC (2017). Parental reflective functioning: theory, Research, and clinical applications. Psychoanal Study Child.

[8] Morón-Duarte LS, Ramirez Varela A, Segura O, Freitas da Silveira M (2019). Quality assessment indicators in antenatal care worldwide: a systematic review. Int J Qual Health Care.

[9] Michelsen SI, Kastanje M, Flachs EM, Søndergaard G, Biering-Sørensen S, Madsen M, Andersen AMN. Evaluering af de forebyggende børneundersøgelser i almen praksis. 2007. https://www.sdu.dk/da/sif/rapporter/2007/evaluering_af_de_forebyggende_boerneundersoegelser_i_almen_praksis. Accessed 16 Apr 2023.

[10] Lykke K, Koefoed P, Håkansson A (2005). Den forebyggende børneundersøgelse - hvad gør vi?. Ugeskr Laeger.

[11] Poulsen ABC. Vejledning om forebyggende sundhedsydelser til børn og unge. 3rd ed. Sundhedsstyrelsen; 2019.

[12] Lähdepuro A, Lahti-Pulkkinen M, Pyhälä R, Tuovinen S, Lahti J, Heinonen K, et al. Positive maternal mental health during pregnancy and mental and behavioral disorders in children: a prospective pregnancy cohort study. J Child Psychol Psychiatry Allied Discip. 2023 May;64(5):807–16. 10.1111/jcpp.13625 35524467

[13] López-López JA, Davies SR, Caldwell DM, Churchill R, Peters TJ, Tallon D (2019). The process and delivery of CBT for depression in adults: a systematic review and network meta-analysis. Psychol Med.

[14] Ma L, Huang C, Tao R, Cui Z, Schluter P (2021). Meta-analytic review of online guided self-help interventions for depressive symptoms among college students. Internet Interv.

[15] Bak PL, Midgley N, Zhu JL, Wistoft K, Obel C (2015). The Resilience Program: preliminary evaluation of a mentalization-based education program. Front Psychol.

[16] Gritt Overbeck JK, Mette Gørtz S, de Voss A, Graungaard IS, Rasmussen. Phil Wilson. Family Wellbeing in General Practice: A study protocol for a cluster-randomised trial of the web-based Resilience Programme on Early Child Development, 06 December 2022, PREPRINT (Version 1). 2022. 10.1186/s13063-022-07045-7 PMC9810520 36597136

[17] Sørensen ER, Overbeck IS, Siersma G, Appel VD, Wilson CL. P. Uptake of signposting to web-based resources: pregnant women’s use of a preventive e-health intervention. 2023 (unpublished data). 10.1186/s12875-023-02130-5 PMC10504765 37716967

[18] May CR, Mair F, Finch T, MacFarlane A, Dowrick C, Treweek S (2009). Development of a theory of implementation and integration: normalization process theory. Implement Sci.

[19] Zigmond AS, Snaith RP (1983). The hospital anxiety and depression scale. Acta Psychiatr Scand.

[20] Felitti VJ (1998). Relationship of childhood abuse and household dysfunction to many of the leading causes of death in adults. The adverse childhood experiences (ACE) study. Am J Prev Med.

[21] Health. U.K.D.o. Recent Life Events Questionnaire. Available from: https://proceduresonline.com/trixcms/media/4898/recent-life-event-questionnaire.pdf. Accessed 25 Apr 2023.

[22] Cresswell K, Sheikh A (2013). Organizational issues in the implementation and adoption of health information technology innovations: an interpretative review. Int J Med Inform.

[23] Gagnon MP, Desmartis M, Labrecque M, Car J, Pagliari C, Pluye P (2012). Systematic review of factors influencing the adoption of information and communication technologies by healthcare professionals. J Med Syst.

[24] Wilhelmsen M, Høifødt RS, Kolstrup N, Waterloo K, Eisemann M, Chenhall R (2014). Norwegian general practitioners’ perspectives on implementation of a guided web-based cognitive behavioral therapy for depression: a qualitative study. J Med Internet Res.

[25] Ludwick DA, Doucette J (2009). Adopting electronic medical records in primary care: lessons learned from health information systems implementation experience in seven countries. Int J Med Inform.

[26] Correa VC, Lugo-Agudelo LH, Aguirre-Acevedo DC, Contreras JAP, Borrero AMP, Patiño-Lugo DF (2020). Individual, health system, and contextual barriers and facilitators for the implementation of clinical practice guidelines: a systematic metareview. Health Res Policy Syst.

[27] Yusof MM, Stergioulas L, Zugic J (2007). Health information systems adoption: findings from a systematic review. Stud Health Technol Inform.

[28] Greenhalgh T, Robert G, Macfarlane F, Bate P, Kyriakidou O (2004). Diffusion of innovations in service organizations: systematic review and recommendations. Milbank Q.

[29] Kelders SM, Kok RN, Ossebaard HC, Van Gemert-Pijnen JE (2012). Persuasive system design does matter: a systematic review of adherence to web-based interventions. J Med Internet Res.

[30] Wangberg SC, Bergmo TS, Johnsen JA (2008). Adherence in internet-based interventions. Patient Prefer Adherence.

[31] Yardley L, Morrison LG, Andreou P, Joseph J, Little P (2010). Understanding reactions to an internet-delivered health-care intervention: accommodating user preferences for information provision. BMC Med Inform Decis Mak.

[32] Latulippe K, Hamel C, Giroux D (2017). Social Health Inequalities and eHealth: A literature review with qualitative synthesis of theoretical and empirical studies. J Med Internet Res.

